# Air-bridged Schottky diodes for dynamically tunable millimeter-wave metamaterial phase shifters

**DOI:** 10.1038/s41598-021-85565-z

**Published:** 2021-03-16

**Authors:** Evangelos Vassos, James Churm, Jeff Powell, Colin Viegas, Byron Alderman, Alexandros Feresidis

**Affiliations:** 1 Department of Electrical, Electronic and Systems Engineering, University of Birmingham, Birmingham, B15 2SA UK; 2 Teratech Components Ltd., STFC, Rutherford Appleton Laboratory, Harwell, Oxford, OX11 0QX UK

**Keywords:** Electrical and electronic engineering, Electronics, photonics and device physics

## Abstract

A low loss metamaterial unit cell is presented with an integrated GaAs air-bridged Schottky diode to produce a dynamically tunable reflective phase shifter that is capable of up to 250° phase shift with an experimentally measured average loss of 6.2 dB at V-band. The air-bridged Schottky diode provides a tuneable capacitance in the range between 30 and 50 fF under an applied reverse voltage bias. This can be used to alter the resonant frequency and phase response of a split patch unit cell of a periodic metasurface. The air-bridged diode die, which is flip-chip soldered to the patch, has ultra-low parasitic capacitance and resistance. Simulated and measured results are presented which verify the potential for the attainment of diode switching speeds with acceptable losses at mmWave frequencies. Furthermore the study shows that this diode-based unit cell can be integrated into metamaterial components, which have potential applications in future mmWave antenna beam-steering, intelligent reflecting surfaces for 6G communications, reflect-arrays, transmit-arrays or holographic antennas.

## Introduction

For more than 50 years, diodes have been a key technology in radio frequency and microwave engineering, enabling the utility and ubiquity of modern tuneable communication systems. Diode-based tuning enables the development of phase shifting components, which are used across the telecommunications industry to produce the phased array antenna systems^1^ that are a necessity for modern wireless communications. However, as frequency increases, parasitic elements within a diode, such as the pad-to-pad capacitance, limit the loss performance of conventional radio frequency diodes, especially when considering their use within the millimetre-wave (mmWave) spectrum and beyond. As emerging applications such as 5G/6G and satellite communications^2, 3^ begin to explore the use of the mmWaves, there is a large body of research being established in different tunable materials and devices for use at these frequencies. These include liquid crystals^4, 5^, ferroelectric substrates^6^, microelectromechanical systems^7–9^, vanadium dioxide^10, 11^, graphene^12, 13^ and piezoelectric actuators^14, 15^. Each of these technologies have their own merits and drawbacks, in terms of switching speed, ease of fabrication, reliability and loss performance^16^.

Diode technology at mmWave frequencies and above, aims to minimise parasitic circuit elements which degrade the high frequency performance^17^. There is significant interest in the use of novel diode technologies, such as those based on Gallium Arsenide (GaAs) air-bridged Schottky configurations, which are designed to remove significant parasitic elements that limit high frequency performance^18^. Applications of this class of GaAs Schottky diodes are found in mixers and frequency multipliers at submillimetre frequencies for use in test equipment, scientific experimentation, security and communication system components up to and beyond 300 GHz^19, 20^. The air-bridged Schottky diode configuration reduces the anode to cathode capacitance by removing the GaAs between mesas, with a gold finger contact bridging the gap between the two halves of the device^21^. Epitaxy design, and anode area is optimised to maximise the junction capacitance change with applied bias. The switching speed for these devices is typically less than a picosecond, with their limit set by biasing circuitry, instead of the tuneable material itself.

Metamaterials are another emerging technology that has been in development for nearly 20 years^22, 23^. Metamaterial systems continue to be developed from microwave to optical frequencies^5, 11^. At microwave bands, tuneable metasurfaces have shown significant promise in the development of exotic communication and sensor functions such as transmit- and reflect-arrays^24–29^, high impedance surfaces^16^, anomalous reflection^30^, absorption^5, 10, 31^ and anisotropic polarisation conversion^32^, along with propagating mode to surface mode conversion^33^. Metasurfaces act as bi-dimensional structures that can modify incident electromagnetic waves in ways that are not possible using classical materials^34^. This unusual interaction with waves is made possible due to the composition of a metasurface, which is made up of an array of periodic or aperiodic unit cells that contain two or more materials with sufficiently contrasting electric properties, so that they can be arranged in ways that force the desired resultant wave behaviour^35^. Metamaterials and metasurfaces have been revolutionising electromagnetics and have applications in many antenna and sensing systems^34–36^.

Tuneable metasurfaces have attracted significant research interest in recent years, with systems working below 10 GHz using either PIN diodes to switch between resonant elements^37^ or varactor diodes to selectively load elements to affect their tuning^38^. However, at higher frequencies performance becomes limited by losses and device parasitic elements.

Phase shifters are important components in many types of systems, including phased-array antennas which are of considerable interest for modern communication applications. Fast operation and broad bandwidth are usually required in phase shifting elements whether they are operating within phased array or metamaterial systems. In the metamaterial context, it is also desirable that the phase shift of an individual unit cell element can be selectively modified to provide maximum reconfigurability (as opposed to addressing the entire array at once). In metamaterial systems this would enable versatile reconfigurable reflect-array and transmit-array technology as well as holographic surfaces^24, 38–43^. With the use of GaAs air-bridged Schottky diodes in each unit cell, this level of reconfigurability within a metasurface may be achievable.

In this paper, a phase shifting unit cell is presented that utilises a GaAs air-bridged Schottky diode to produce a reflection phase shift at 41.5 GHz. Performance has been optimised for large phase shift, low loss and manufacturability. In order to demonstrate the validity of this design, a unit cell prototype embedded within a waveguide was designed at 48 GHz and measured. There is a frequency shift between simulated and measured systems due to the tolerances of the quartz dielectric and waveguide, in all other ways the model behaves within expectations and demonstrates the viability of air-bridged Schottky diodes as tuning elements in mmWave systems. The unit cell demonstrated in this study provides supporting evidence that large scale, low loss, individually addressable, reconfigurable metasurfaces may be possible at switching speeds that are limited by control line parasitics, rather than the physical limits of the switching element itself^20^.

## Results

### Unit cell and structure

**Figure 1:**
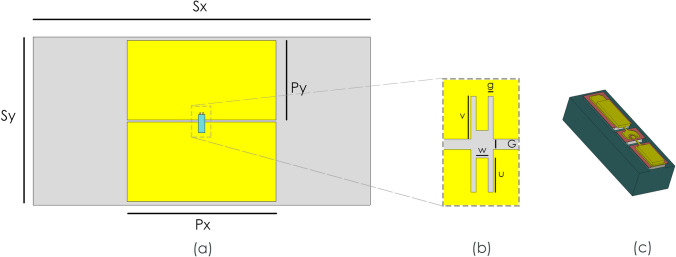
A schematic of an air bridged Schottky diode controlled phase shifting unit cell. (**a**) Schematic of the front view of the proposed unit cell, with quartz thickness 0.2 mm with ground plane backing. (**b**) The diode bonding site, designed to have minimal effect on EM performance, whilst allowing for flip-chip bonding of the diode. (**c**) The full 3D model of the GaAs diode.

A significant advantage in periodic metasurface design is that the performance of a sufficiently large metasurface can be extracted from the response of an individual unit cell when modelled with periodic boundary conditions. This is because periodic boundaries emulate the electromagnetic response of an infinite sheet of repeated unit cells^36^. The unit cell presented here consists of two thin film rectangular gold patches, printed on a fused silica (quartz) substrate, with a gold back side ground plane. This unit cell exhibits an engineered reflection phase that can be controlled by the dimensions or resonance of the patches. Bridging these two patches is a GaAs air-bridged Schottky diode, as shown in Fig. 1a. By varying an applied reverse bias to the diode, the junction capacitance also varies and this shifts the resonance of the bridged patch to produce a tunable reflective phase shifting unit cell. Obtaining a phase shifter using this system is a delicate trade-off between losses and tunability, with patch parameters needing to be designed alongside accurate simulation of the diode.

The full model of the diode used in simulations can be seen in Fig. 1c. The voltage independent diode parasitics are captured through the use of the complete 3D diode model in the electromagnetic simulations. By the application of a reverse bias, the magnitude of junction capacitance can be altered between 30 and 50 fF which enables the unit cell to produce the phase shifting effect. The junction resistance is the main source of loss within the system. The total diode resistance is typically between 1 and 5 Ω; this value varies only slightly with junction voltage and so is considered to be a constant in simulations. The value of the junction resistance is critical to the loss performance of the overall system.

### Dynamic phase tuning high impedance surface

**Figure 2:**
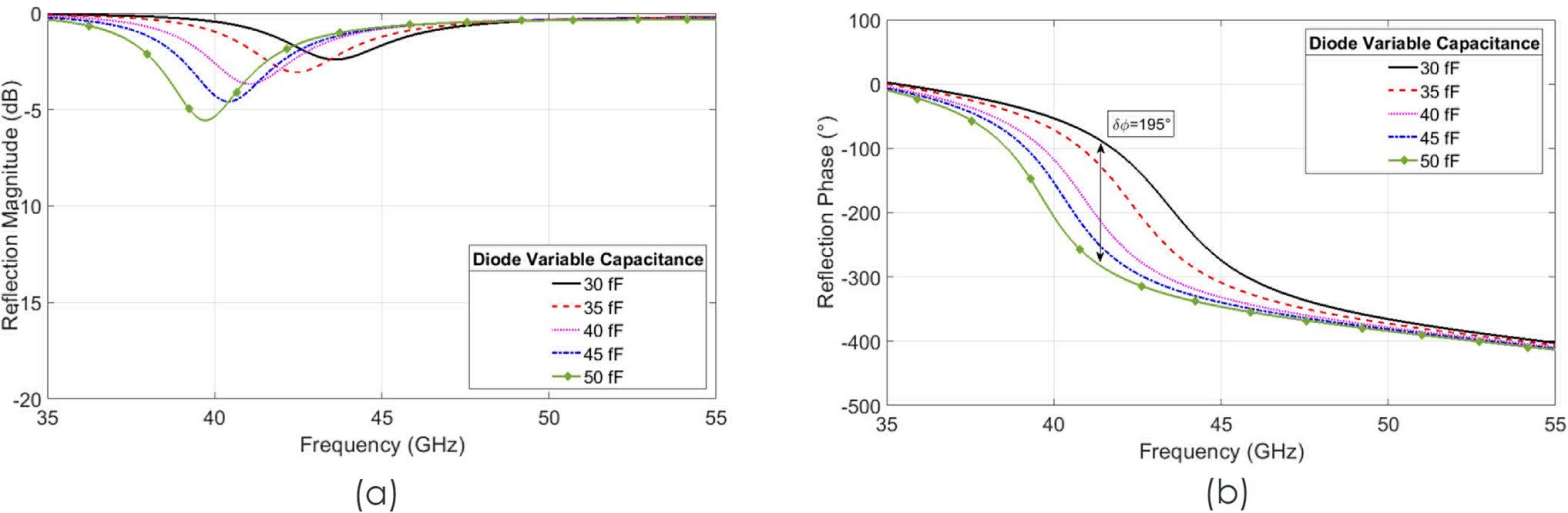
Simulation results of the proposed periodic unit cell array. (**a**) Reflection magnitude simulation results with varying junction capacitance. (**b**) Reflection phase simulation results of the unit cell with varying junction capacitance.

The surface layout of the proposed unit cell can be seen in Fig. 1, with dimensions as given in Table 1, with a junction resistance of 5 Ω and a capacitance variation from 30 to 50 fF. This design provides phase shift at a central frequency of around 41.5 GHz. The thicknesses of the quartz substrate and gold metallisation are 0.20 mm and 1 µm respectively. In order to demonstrate what a high impedance surface comprised of such unit cells would yield, a full periodic simulation was performed with a full 3D model of the diode geometry (Fig. 1c) included in order to capture all parasitic elements introduced by the inclusion of the diode. The junction resistance and capacitance were set by the introduction of a small gap between the anode contact and the GaAs junction, which was shorted by a lumped element model with variable resistance and capacitance. For this simulation the junction resistance was set to 5 Ω and the capacitance varied to provide a reflected phase shift which can be seen in Fig. 2. These simulated results demonstrate that a maximum phase shift of 195° at around 41.5 GHz can be achieved with average losses of 2.5 dB, when using a periodic array of the unit cells. When paired with the nano-second switching speed enabled by GaAs air-bridged Schottky diodes, this demonstrates an unprecedented combination of low loss phase shift and switching speed^19^.

**Table 1:** Unit cell dimensions.

Sy	Sx	Py	Px	G	w	v	u	g
**Unit cell dimensions (mm)**								
1.900	3.800	0.930	1.680	0.020	0.024	0.085	0.068	0.010

If the junction resistance of the model were reduced to 1 Ω, this would further improve average loss performance from 2.5 to 1.0 dB with the same phase shift of 195°. Typical diode parameters can be seen in Table 2. The resulting structure is very sensitive to small parameter changes, for example, a change in the thickness of the substrate from 0.20 to 0.16 mm changes the tuning frequency by 3.5 GHz; in addition the same variation increases the average reflective loss to approximately 3.5 dB with a maximum phase shift 210° at 41.5 GHz. With the materials used, a 20% uncertainty in substrate thickness could be expected. Due to this, a compromise was reached between performance and ensured manufacturability, which is reflected in the final values as in Table 1.

**Table 2:** Diodes parameters.

	Chip length (µm)	Chip width (µm)	Chip thickness (µm)
**Manufactured diodes parameters**			
Min	326	46	45
Typ	336	56	50
Max	346	66	55

Air-bridged Schottky diodes parameters including the dimensions of the chip and the diodes characteristic values as measured by the manufacturer.

Additionally, the structure was studied for the durability of its performance as the angle of incidence (θ and φ) changes. The angle θ is defined by the inclination of the incident electromagnetic wave with respect to the z axis and the angle φ which defines the rotation of the electromagnetic wave at the xy plane. For the change of the angle θ from normal incidence to 30°, the increase of the phase shift is observed to around 200° with simultaneous increase of the average losses to 2 dB. The angular stability study of the angle φ suggests that a change from normal incidence to 15° maintains an average loss of about 2.5 dB while obtaining a maximum phase shift of 180°.

### Experimental verification

**Figure 3:**
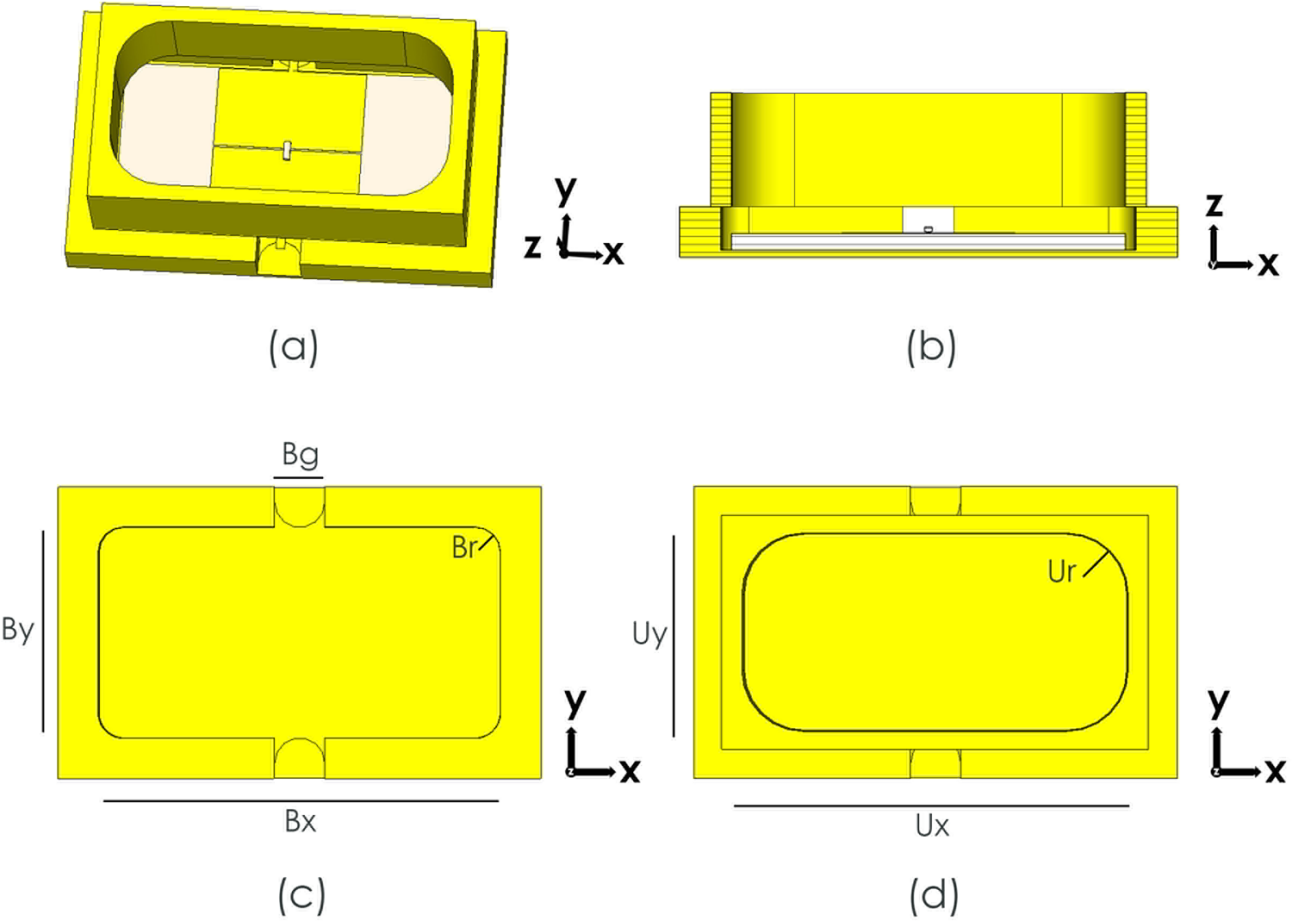
Schematic of the proposed unit cell and waveguide (**a**) Perspective view of the unit cell inside the waveguide. (**b**) Cutting plane y of the proposed structure. (**c**) View of the waveguide short backing where By = 2.00 mm, Bg = 0.50 mm Bx = 4.00 mm and Br = 0.25 mm. (**d**) Front view of the top waveguide where Uy = 1.95 mm, Ux = 3.80 mm and Ur = 0.60 mm.

**Figure 4:**
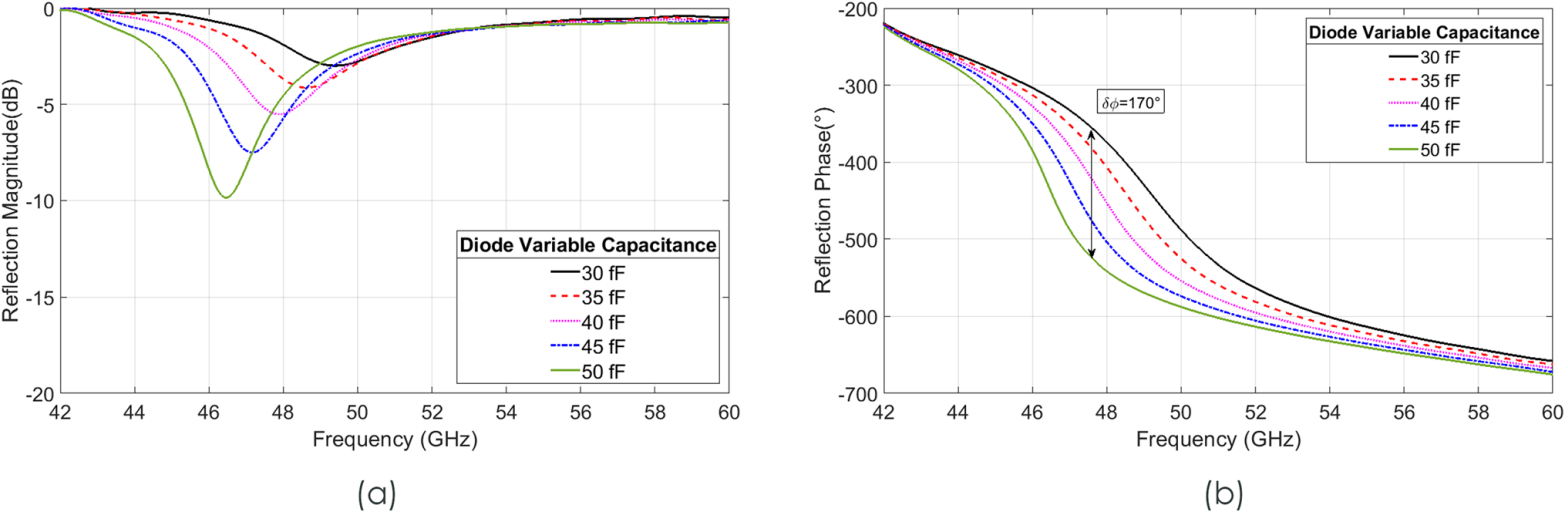
Simulation results of the isolated unit cell inside a waveguide (**a**) Reflection magnitude simulation results with varying the junction capacitance. (**b**) Reflection phase simulation results of the unit cell with varying the junction capacitance.

As the fabrication complexity of a full metasurface containing GaAs air-bridged Schottky diodes would have been prohibitive at this early stage of investigation, to obtain a proof of principle, a prototype unit cell was produced at the end of a shorted rectangular waveguide section so that a single unit cell could be simulated and measured. The unit cell was mounted to a bespoke WR19 waveguide short and subjected to incident TE waves, swept in simulation between 42 and 60 GHz. Fig. 3 shows the simulated waveguide structure with small holes in the middle of the long sides of the waveguide visible in Fig. 3b, through which biasing lines could be passed. The results of this simulation, presented in Fig. 4, show a maximum phase shift of 170° at 47.6 GHz with average losses of 4.3 dB. There is a deviation here from the unit cell periodic simulation due to the presence of a small air gap (0.1 mm) around the unit cell, within the waveguide. This gap is required due to the manufacturing and tolerance of both waveguide enclosure and the quartz substrate, and results in the 6.1 GHz shift between periodic and waveguide bound simulations.

The biasing holes in the walls of the waveguide, were designed so that their dimensions are much smaller than the operating wavelength. The result is the maintenance of field distribution despite their presence. Simulations suggest that the introduction of these two holes slightly affects the operating frequency of the unit cell by shifting it from 48.3 GHz to 47.6 GHz, without affecting losses or phase shift.

A full prototype of the waveguide system was fabricated and assembled by Teratech Ltd. and mounted into a CNC machined waveguide short. Two devices were fabricated so the effect of the loaded unit cell could be isolated from imperfections in the waveguide structure. The first measurement was of an empty waveguide short section and the second was a waveguide shorted section with a full device mounted at its end. The losses obtained in the measurement of the empty waveguide were removed from those of the full device in order to remove the effects of surface roughness and tolerance issues within the waveguide itself. This was done to isolate the performance of the unit cell under investigation.

**Figure 5:**
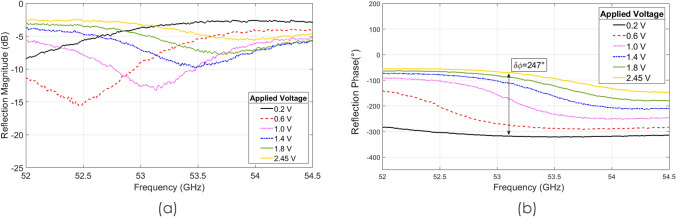
Measured results of the prototype unit cell (**a**) Reflection magnitude measured results with varying voltage. (**b**) Reflected phase shift simulation results of the unit cell with varying voltage**.**

Measurements for the full device were taken at varying bias voltages. By removing the losses introduced by the empty waveguide section, it was possible to assess the performance of the unit cell, and the results are shown in Fig. 5. This shows that a maximum phase shift of around 250° is achievable at 53.1 GHz with a 180° phase shift defining the operational bandwidth between 53.1 and 54.1 GHz (1 GHz bandwidth), with average loss of 6.2 dB. Minimum average losses of 5.1 dB can be achieved at 54.1 GHz with a phase shift of 180°.

## Discussion

**Figure 6:**
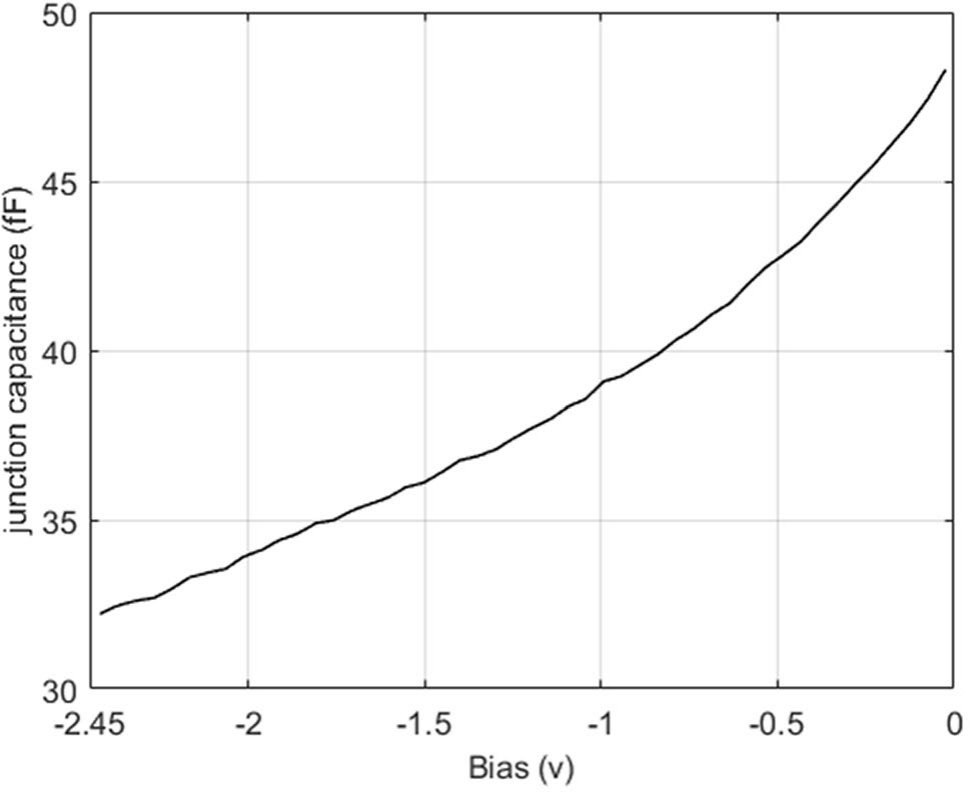
Measured capacitance–voltage characteristic. The C–V characteristic of the diode used for this experiment**.**

The switching speed, potential architecture flexibility, achievable phase shift and loss performance make GaAs air-bridge Schottky diode enabled metasurfaces one of the most competitive technologies where there is a requirement for fully electronic fast switching speed at high frequencies. Typically the use of diodes at mmWave frequencies have been limited by high losses^44^, however, the work presented here demonstrates that the integration of state-of-the-art diodes into metasurface designs can yield low loss diode based phase shifters at high frequencies. The air-bridged Schottky diodes measured C-V characteristic is shown in Fig. 6.

Discrepancies between the simulated and measured unit cell results are observed in the central operating frequency, in the value of the losses and of the phase shift obtained. These discrepancies are mainly attributed to the uncertainty in quartz thickness, as parameter sensitivity studies (shown in Table 3) show that a decrease in the target thickness of the quartz increases the average resonant frequencies along with adding additional losses. Any extra losses unaccounted for by substrate thickness can be attributed to slight errors in parameter *g* in Fig. 2b and differences in the solder joint model versus reality.

**Table 3:** Structures sensitivity study.

Parameter	Target value	Units	Min value	Max value	Effective frequency centre range (GHz)	Effective average loss range (dB)
R	5	Ω	1	5	47.6	2.6–5.6
Py	0.93	mm	0.925	0.935	47.9–48.3	5.6
G	0.02	mm	0.01	0.03	46.5–47.9	4.0–7.1
St	0.20	mm	0.16	0.24	50.4–45.6	7.9–4.7
By	2.00	mm	1.95	2.10	45.9–48.0	6.3–5.6

Sensitivity study of the effect of key parameters on the performance of the structure.

Table 4 presents the key performance characteristics of the proposed technology in comparison to other technologies used in tuneable phase shifting systems. Direct comparisons were not always available; however it can be seen that air-bridged Schottky diodes average losses are competitive, but that their switching speeds can be orders of magnitude faster than other state-of-the-art offerings. The switching speed of the diode was not measured in this study, and a conservative estimate of < 10 ns has been chosen as a comparison.

**Table 4:** The performance characteristics of the most well-known V-band tuning methods when used in periodic metasurface based structures.

Technology^ref^	Frequency range (GHz)	Application	Switch speed	Reflection phase shift (°)	Reflection average losses (dB)	Individually addressable unit cell
Air bridge Schottky diodes (this work)	53.1–54.1	Phase shifting unit cell	< 10 ns	250	6.2	Yes
Piezoelectric actuators^14^	35.0–65.0	Phase shifting surfaces	~ 2 ms	180	1.0	No
	57.0–62.0		~ 2 ms	360	1.8	No
Liquid crystals^45^	76.0–78.0	Reflectarray	–	280	6.7	Yes
Liquid crystals^46^	26.6–27.7	Fabry–Perot resonator	> 20 ms	N/A	N/A	Yes
Ferroelectric^47^	19.0	Reflectarray	~ sub-μsec	320	7.5	Yes

Indicative values for the main operating characteristics such as the frequency operating range, the switching speed, the reflection phase shift, the reflection average losses and the possibility of controlling individually the unit cells.

With this contribution a promising phase shifting unit cell has been designed that can enable individually addressable phase shifting elements of a metasurface to be produced within millimetre wave frequencies. To demonstrate this tuning method, a unit cell has been designed in a waveguide test structure. Although there are frequency shifts between both, periodic and waveguide simulations, and simulated and measured data (which are explained by practical necessities of the measurement method), the data suggests that competitive phase shifting performance can be obtained with switching speeds that are far superior to other competing technologies.

In addition to the natural suitability of the proposed tuning technique to holographic antennas as well as reconfigurable reflect- and transmit-arrays, the proposed structure can be used as reconfigurable high impedance surfaces that can act as the ground planes of tuneable antenna or cavities. Along with this, leaky wave antenna applications could provide highly directional beam scanning antenna using this tuning technology. This configuration provides a much lower loss system than other diode-based technologies permit. Furthermore, the nature of the solution would enable high density, individually addressable diodes to be included within a metasurface, offering a potentially viable way forward for the creation of fast-switching holographic antennas at mmWave and above.

## Methods

The design and simulation of the unit cell presented within this work were produced using full wave simulation software (CST Microwave Studio), with the single unit cell being simulated with periodic boundary conditions and the waveguide integrated unit cell within a full metal waveguide housing. The Schottky diode was modelled in full between the two patches and values of the junction capacitance and resistance embedded within the physical model of the diode using a variable lumped element. The inclusion of a full detailed physical model of the diode into the simulation software meant that other parasitic parameters within the diode were automatically captured in the simulated results and therefore the design was optimised with these already in place.

The Schottky diode used in this experiment includes a channel etched into the substrate, between the anode and cathode contacts which is then bridged by a metallic finger, which reduces the parasitic capacitance. Resulting in a reduction in total capacitance for the device^21^. The diode was fabricated as a AS3/4G2/31p6 diode available from Teratech Ltd., and cleaved via dicing saw by the company to provide a single junction device.

The unit cell was created using standard photolithography and wet etch techniques on a quartz substrate. A common commercially available thickness of quartz is 0.10 ± 0.02 mm. In order to build this up to the 0.20 mm required for this study, two substrates were diced to the desired dimensions. The top fragment was coated in gold using standard sputter coating techniques, and underwent photolithography, then wet etch to produce the unit cell patch design with a gap ready for the placement of the diode. The prefabricated and cleaved diode was flip-chip bonded using indium solder to the bridge site between the two patches of the unit cell. The back substrate had a gold ground plane sputter coated onto the back side. The two substrates were then aligned within the waveguide and fixed to each other using a thin film of non-conductive epoxy resin with thickness less than 1 μm.

**Figure 7:**
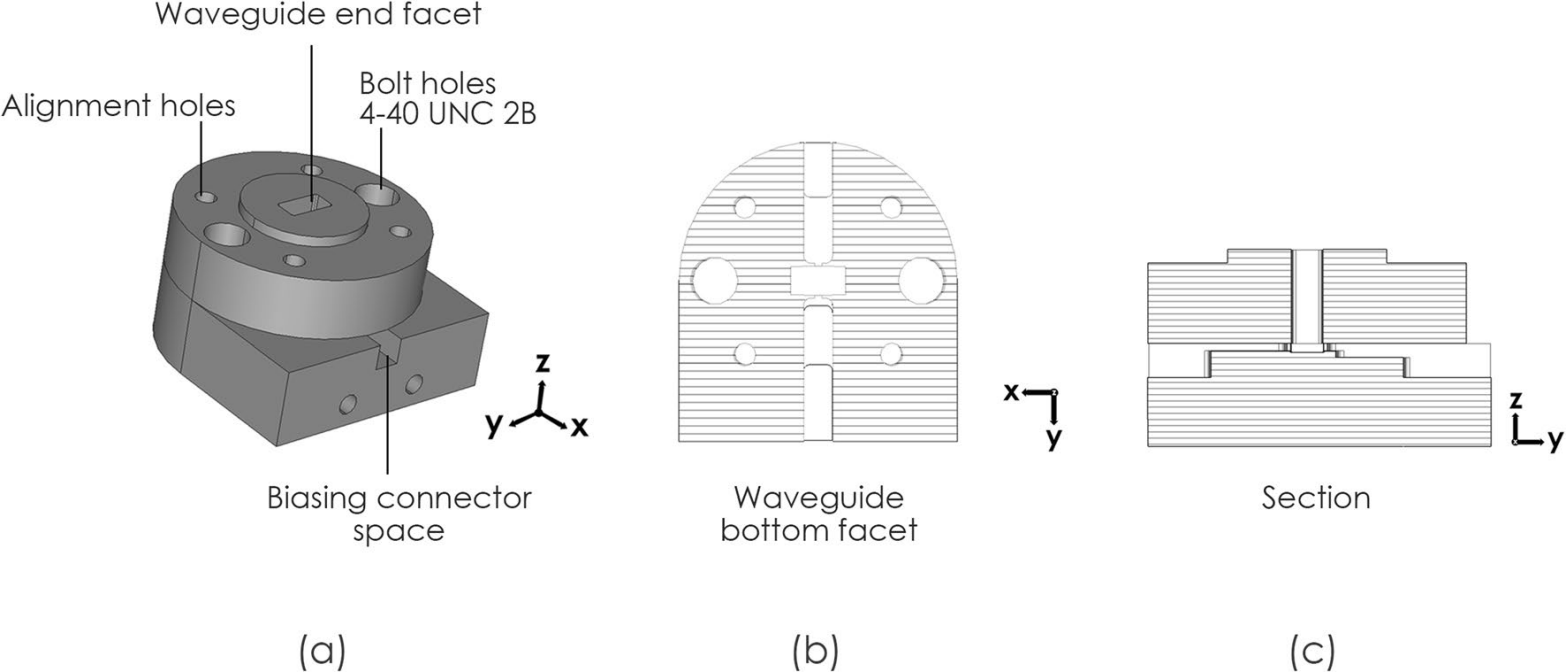
Schematic of the proposed waveguide (**a**) perspective view of the waveguide. (**b**) Cutting plane z and (**c**) cutting plane x of the proposed structure.

**Figure 8:**
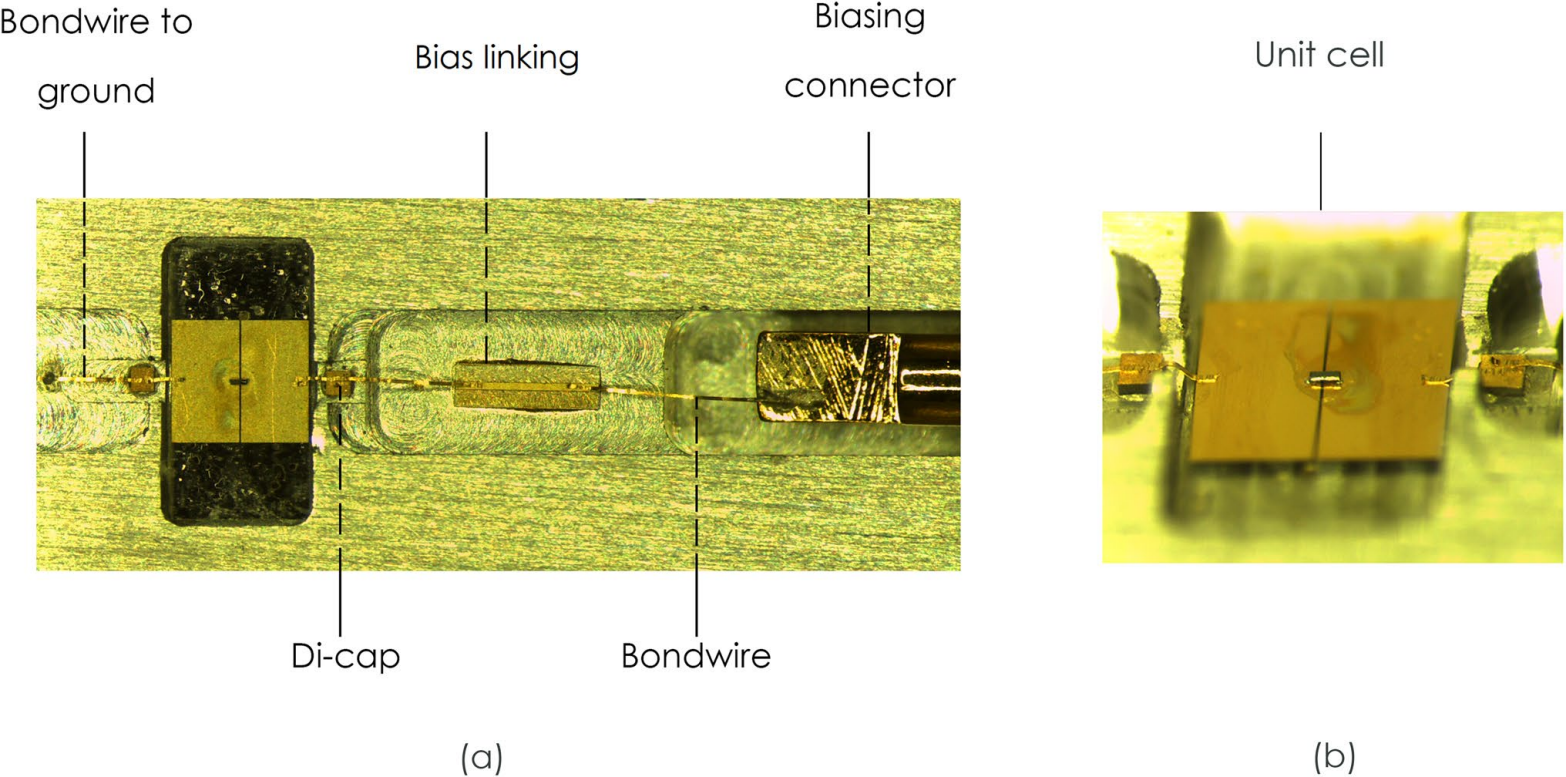
Photograph of fabricated metasurface unit cell inside the waveguide. (**a**) The photograph highlights the biasing supply circuit and (**b**) side view of the unit cell utilising an air-bridged Schottky diode.

The bespoke WR19 waveguide short was designed to accommodate the unit cell, with minimum tolerance (of ± 20 μm) around the substrate and space for biasing lead access, along with housing for the small biasing circuitry which consisted of a small di-cap bonding site and bias connection. The edges of the unit cell patches were wire-bonded through a gap in the waveguide wall to the di-cap. The grounded side was bonded to the metal housing of the waveguide and the side where bias voltage would be applied was wire bonded to a small biasing line and then onto the biasing connector. The schematic of the waveguide short can be seen in Fig. 7, with the internal assembly of the biasing components in Fig. 8a and the unit cell shown in Fig. 8b.

The device configuration is currently very sensitive to minor errors in fabrication, and as such parameter studies were required in order to choose parameters best suited, and most resistant, to standard fabrication tolerances. The results of the parameter studies can be seen in Table 3.

For the measurements a ZVA65-A VNA was used to measure S-parameters for the waveguide short as a bias voltage was applied. A standard single port calibration was preformed prior to the study to remove any undesired effects of the measurement setup.
